# Self-Reported Neuropsychiatric Post–COVID-19 Condition and CSF Markers of Neuroinflammation

**DOI:** 10.1001/jamanetworkopen.2023.42741

**Published:** 2023-11-10

**Authors:** Shelli F. Farhadian, Hailey D. Reisert, Lindsay McAlpine, Jennifer Chiarella, Priya Kosana, Jennifer Yoon, Serena Spudich

**Affiliations:** 1 Section of Infectious Diseases, Yale School of Medicine, New Haven, Connecticut; 2 Department of Epidemiology of Microbial Diseases, Yale School of Public Health, New Haven, Connecticut; 3 Department of Neurology, Yale School of Medicine, New Haven, Connecticut; 4 Center for Brain and Mind Health, Yale University, New Haven, Connecticut

## Abstract

This case-control study assesses cerebrospinal fluid markers of neuroinflammation and blood-brain barrier disruption in individuals with post–COVID-19 condition who reported neuropsychiatric symptoms.

## Introduction

Long COVID, also known as post–COVID-19 condition (PCC), refers to a range of symptoms that may persist for weeks to months after acute COVID-19. A subset of people with PCC report neuropsychiatric symptoms (neuro-PCC); however, the mechanisms behind this phenomenon are not yet understood.^1^ Cerebrospinal fluid (CSF) circulates throughout the central nervous system, serving as a window to the brain and providing a means to study neuropathology in living people.^2,3,4^ In this study, we assessed for CSF markers of neuroinflammation, including microglial activation, and blood-brain-barrier disruption in individuals with neuro-PCC and individuals who have never had COVID-19.

## Methods

This case-control study was approved by the institutional review board at Yale University. All participants provided written informed consent. This study followed reporting requirements of the Strengthening the Reporting of Observational Studies in Epidemiology (STROBE) reporting guideline.

Participants with neuro-PCC were enrolled in The COVID Mind Study at Yale University if they self-reported new or worsened neuropsychiatric symptoms at least 3 months after laboratory–confirmed COVID-19. The control group included asymptomatic participants who were recruited before the COVID-19 pandemic (prior to 2020) to serve as controls for other studies and included 1 asymptomatic participant who was enrolled in 2022 and had no history of COVID-19 illness, which was supported by a negative nucleocapsid antibody in the blood. Participants were excluded if they had any history of severe neurological or psychiatric illness, severe immunocompromising condition, or were receiving immune-suppressive medications at the time of the study. Information regarding the date of positive SARS-COV-2 polymerase chain reaction (PCR) test and vaccination was obtained through interviews and medical record review. All participants consented to large-volume lumbar puncture (up to 30 cc CSF removed) and blood draw for research purposes.

Frozen CSF supernatant and plasma from all participants were profiled using a 15-plex cytokine laser bead assay. Neopterin was measured by commercially available enzyme-linked immunosorbent assay.

For demographic and clinical data, group comparisons were made using *t* tests, except for race where group comparisons for race were performed using the 2-proportion *z* test. Race and ethnicity were assessed through self-report. For cytokine data, participants with neuro-PCC and control participants who had never had COVID-19 were compared using nonparametric unpaired multiple Mann-Whitney tests. The false discovery rate (FDR) was controlled using the Benjamini, Krieger, and Yekutieli 2-stage step-up method. Statistical tests were computed using R statistical software version 4.2.3 (R Project for Statistical Computing). Statistical significance was set at *P* < .05 after adjusting for multiple comparisons.

## Results

All 37 participants with neuro-PCC (median [IQR] age, 48 [38-60] years; 4 [10.8%] Black or African American individuals; 29 [78.4%] White individuals; 27 [73.0%] females) had prior laboratory confirmed COVID-19 with a known date of SARS-CoV-2 PCR test positivity that occurred between March 2020 and July 2022. Twenty-two control participants were included in the study (median [IQR] age, 51.5 [38.8-56.8] years; 6 [27.3%] Hispanic individuals; 10 [45.5%] Black or African American individuals; 7 [31.8%] females). Most participants with neuro-PCC (29 of 37 [78%]) had acute COVID-19 at times when SARS-CoV-2 Alpha variant was the dominant circulating strain in the US. Brain fog and cognitive impairment (31 [84%]) and excessive fatigue (31 [84%]) were the most frequent post–COVID-19 symptoms reported in the neuro-PCC group. Demographic and clinical characteristics of the cohort are reported in Table 1.

**Table 1.** Demographics and Clinical Characteristics of Study Participants^a^

Characteristics	Patients, No. (%)		*P* value
	Neuro-PCC (n = 37)	Controls (n = 22)	
Age, median (IQR)	48 (38-60)	51 (39-57)	.88
Race or ethnicity			
Asian	0	1 (4.5)	.19
Black or African American	4 (10.8)	10 (45.5)	.002
Hispanic	4 (10.8)	6 (27.3)	.10
White	29 (78.4)	5 (22.7)	<.001
Gender			
Female	27 (73.0)	7 (31.8)	.002
Male	10 (27.0)	15 (68.2)	
Years of education, median (IQR)	16 (13.75-17.25)	12 (12-15.5)	.03
BMI, median (IQR)	29.03 (26.21-36.21)	27.30 (25.23-30.97)	.11
Comorbiditities			
Alcohol	0	7 (31.8)	<.001
Smoking	2 (5.4)	13 (59.1)	<.001
Hypertension	10 (27.0)	4 (18.2)	.45
Type 2 diabetes	3 (8.1)	3 (13.6)	.50
Obesity	7 (18.9)	1 (4.5)	.12
Antidepressant use within last 12 mos	16 (43.2)	3 (13.6)	.02
Acute COVID-19 course			
Days between COVID-19 diagnosis and research lumbar puncture, median (IQR)	329 (268-430)	NA	NA
Highest level of care			
Home	29 (78.4)	NA	NA
Hospital floor	7 (18.9)	NA	NA
ICU	3 (8.1)	NA	NA
Vaccination status at time of infection			
Fully vaccinated	4 (10.8)	NA	NA
Unvaccinated	33 (89.2)	NA	NA
Vaccination status at time of PCC research study visit			
Fully vaccinated	16 (43.2)	NA	NA
Partially vaccinated	1 (2.7)	NA	NA
Unvaccinated	10 (27.0)	NA	NA

Abbreviations: BMI, body mass index (calculated as weight in kilograms divided by height in meters squared); ICU, intensive care unit; NA, not applicable.

^a^ Group comparisons were made using *t* tests, except for race where group comparisons for race were performed using the 2-proportion *z* test.

CSF and plasma inflammatory measures are reported in Table 2. The CSF white blood cell count and protein levels were not elevated in the neuro-PCC group, nor was the CSF to blood albumin ratio, which is altered under conditions of blood-brain barrier breakdown. CSF IgG index and a comparison of oligoclonal bands in the CSF and blood did not reveal evidence for intrathecal immunoglobulin production.

**Table 2.** CSF and Plasma Inflammatory Markers Among Participants With Neuro-PCC and Controls^a^

Analyte/biomarker	CSF, median (IQR)		*P* value for CSF^b^	Blood, median (IQR)		*P* value for blood^b^
	Neuro-PCC	Control		Neuro-PCC	Control	
Clinical CSF Measures						
CSF WBC, cells/uL	1.5 (1.0-3.0)	1.0 (0-3.5)	.60	NA	NA	NA
CSF protein, mg/dL	31.95 (27.5-37.9)	30.20 (24.5-42.75)	.81	NA	NA	NA
Biomarkers/analytes						
GM-CSF, pg/mL	0	0	>.99	0	0	.66
IL-2, pg/mL	0 (0-.003)	0 (0-.005)	>.99	0.01 (0-.87)	0 (0-.06)	.51
IFNγ, pg/mL	0.01 (0-.04)	0.01 (.01-.08)	.19	2.88 (.35-6.7)	1.09 (0-3.3)	.51
IL1B, pg/mL	0	0	>.99	18.28 (6.3-34.7)	8.04 (5.1-16.5)	.51
IL-1Ra, pg/mL	0.04 (.02-.07)	0.04 (.02-.07)	>.99	4.03 (2.3-8.7)	3.01 (1.6-5.7)	.66
IL-4, pg/mL	0 (0-.012)	0 (0-.005)	.21	0.35 (0-1.0)	0.20 (0-.37)	.66
IL-5, pg/mL	0.81 (.66-1.01)	0.81 (.63-1.02)	>.99	4.22 (2.4-7.4)	3.96 (2.3-10.9)	.84
IL-6, pg/mL	1.28 (1.0-1.8)	1.84 (1.2-2.4)	.19	1.66 (1.1-5.3)	1.63 (1.0-3.2)	.75
IL-8, pg/mL	25.19 (22-31)	29.39 (21-35)	.86	2.20 (.97-3.8)	2.72 (1.0-5.2)	.66
IL-10, pg/mL	0.40 (.31-.48)	0.38 (.29-.48)	>.99	0.32 (0-1.1)	0.07 (0-7.1)	.75
IL-12p40, pg/mL	0.66 (.35-1.3)	0.75 (.39-1.3)	>.99	98.97 (69.7-191)	42.69 (25.6-99)	.07
IL-12p70, pg/mL	0	0	>.99	1.54 (0-6.8)	0.25 (0-3.5)	.51
IL-13, pg/mL	0	0	>.99	46.32 (0-141)	37.57 (0-93)	.75
MCP-1, pg/mL	499 (403-612)	698 (440-1002)	.19	352.9 (246-492)	272.5 (230-414)	.66
TNF-α, pg/mL	0.66 (0.55-0.72)	0.55 (0.52-0.64)	.19	39.12 (30-59)	34.29 (28-49)	.51
Neopterin, nmol/L	5.03 (4.4-6.1)	5.23 (4.3-6.3)	>.99	7.82 (6.0-11.2)	7.97 (6.6-9.6)	.84

Abbreviations: CSF, cerebrospinal fluid; GM-CSF, granulocyte-macrophage colony-stimulating factor; IFNγ, interferon gamma; IL, interleukin; MCP, monocyte chemoattractant protein; PCC, post–COVID-19; TNF-α, tumor necrosis factor-α; WBC, white blood cell.

SI conversion factor: To convert WBCs to ×10^9^ per liter, multiply by 0.001.

^a^ Control participants included individuals who had never had COVID-19.

^b^ *P* values for biomarkers or analytes displayed were adjusted for multiple comparisons. Groups were compared using nonparametric unpaired multiple Mann-Whitney tests. The false discovery rate was controlled using the Benjamini, Krieger, and Yekutieli 2-stage step-up method.

In the CSF, tumor necrosis factor-α levels were elevated (0.66 vs 0.55 pg/ul) while monocyte chemoattractant protein-1 and IL-6 levels were lower in participants with neuro-PCC compared with controls (499 pg/ul vs 697 pg/ul and 1.2 pg/ul vs 1.8 pg/ul, respectively). However, these differences were not statistically significant after accounting for multiple comparisons. There were no significant differences in any of the other cytokines or chemokines tested in the CSF or in the plasma. There were also no elevations in levels of neopterin, a marker of microglial cell activation, in participants with neuro-PCC.

## Discussion

When comparing individuals with neuro-PCC with control participants who had never had COVID-19, we found no evidence of overt neuroinflammation (normal CSF cell count, inflammatory cytokines) or blood-brain barrier dysfunction (normal albumin ratio), suggesting that persistent central nervous system immune activation is not a primary driver of neurological long COVID-19. Strengths of the present study include that all participants with neuro-PCC had laboratory-confirmed history of COVID-19 with known date of PCR positivity, and the inclusion of a control group who had never had COVID-19. Limitations of the study include small sample size, discrepancies between the gender and race of cases compared with controls, and higher rates of smoking and alcohol use and lower rates of antidepressant use in the control group, which may have impacted CSF biomarker results.

## Notes

Supplement.
Data Sharing Statement
