# Supplementary material for: Self-Reported Neuropsychiatric Post–COVID-19 Condition and CSF Markers of Neuroinflammation
Source: JAMA Netw Open. 2023 Nov 10;6(11):e2342741. doi: 10.1001/jamanetworkopen.2023.42741 (PMC10638645; doi:10.1001/jamanetworkopen.2023.42741)
Supplement: Supplement. — Data Sharing Statement [file jamanetwopen-e2342741-s001.pdf]

Data Sharing Statement

Farhadian. Self-Reported Neuropsychiatric Post–COVID-19 Condition and CSF Markers of Neuroinflammation. *JAMA Netw Open*. Published November 10, 2023.
doi:10.1001/jamanetworkopen.2023.42741

Data
Data available: Yes
Data types: Deidentified participant data
How to access data: Requests for data should be sent to shelli.farhadian@yale.edu
When available: With publication

Supporting Documents
Document types: None

Additional Information
Who can access the data: researchers whose proposed use of the data has been approved
Types of analyses: for scientific study
Mechanisms of data availability: after approval of a proposal
